# Enhanced Energy Storage Performance in Na_0.5_Bi_0.5_TiO_3_-Based Relaxor Ferroelectric Ceramics via Compositional Tailoring

**DOI:** 10.3390/ma15175881

**Published:** 2022-08-25

**Authors:** Yuleng Jiang, Xiang Niu, Wei Liang, Xiaodong Jian, Hongwei Shi, Feng Li, Yang Zhang, Ting Wang, Weiping Gong, Xiaobo Zhao, Yingbang Yao, Tao Tao, Bo Liang, Shengguo Lu

**Affiliations:** 1 Guangdong Provincial Research Center on Smart Materials and Energy Conversion Devices, Guangdong Provincial Key Laboratory of Functional Soft Condensed Matter, School of Integrated Circuits, School of Materials and Energy, Guangdong University of Technology, Guangzhou 510006, China; 2 Information Materials and Intelligent Sensing Laboratory of Anhui Province, Institutes of Physical Science and Information Technology, Anhui University, Hefei 230601, China; 3 Guangdong Provincial Key Laboratory of Electronic Functional Materials and Devices, Huizhou University, Huizhou 516001, China; 4 School of Integrated Circuits, Guangdong University of Technology, Guangzhou 510006, China; 5 Dongguan South China Design Innovation Institute, Dongguan 523808, China

**Keywords:** Na_0.5_Bi_0.5_TiO_3_, lead-free, relaxor ferroelectrics, compositional tailoring, energy storage

## Abstract

Owing to the high power density, excellent operational stability and fast charge/discharge rate, and environmental friendliness, the lead-free Na_0.5_Bi_0.5_TiO_3_ (NBT)-based relaxor ferroelectrics exhibit great potential in pulsed power capacitors. Herein, novel lead-free (1−x)(0.7Na_0.5_Bi_0.5_TiO_3_-0.3Sr_0.7_Bi_0.2_TiO_3_)-xBi(Mg_0.5_Zr_0.5_)O_3_ (NBT-SBT-xBMZ) relaxor ferroelectric ceramics were successfully fabricated using a solid-state reaction method and designed via compositional tailoring. The microstructure, dielectric properties, ferroelectric properties, and energy storage performance were investigated. The results indicate that appropriate Bi(Mg_0.5_Zr_0.5_)O_3_ content can effectively enhance the relaxor ferroelectric characteristics and improve the dielectric breakdown strength by forming fine grain sizes and diminishing oxygen vacancy concentrations. Therefore, the optimal *W_rec_* of 6.75 J/cm^3^ and a *η* of 79.44% were simultaneously obtained in NBT-SBT-0.15BMZ at 20 °C and 385 kV/cm. Meanwhile, thermal stability (20–180 °C) and frequency stability (1–200 Hz) associated with the ultrafast discharge time of ~49.1 ns were also procured in the same composition, providing a promising material system for applications in power pulse devices.

## 1. Introduction

With the great need for green energy, electrical energy storage has become an important technology to store the energies generated from the wind, solar energy, tide, and other sources [1,2]. Nowadays, numerous energy storage devices, such as batteries, electrochemical bilayer capacitors, and dielectric capacitors, have been extensively investigated in recent decades, with dielectric capacitors being regarded as a more promising category for high power industries owing to their ultrahigh power density, excellent operational stabilities, superior charge-discharge performance (less than 100 ns), fatigue resistance, and impressive mechanical properties [3].

Theoretically, to evaluate the energy storage performance (ESP) of a dielectric material, the energy storage density (W) and energy storage efficiency (η) are two major parameters, which can be deduced based on the polarization-electric field (P-E) hysteresis loops, and expressed as follows [4].

(1) $$W_{total}=\int_{0}^{P_{m}}EdP,$$

(2) $$W_{rec}=\int_{P_{r}}^{P_{m}}EdP,$$

(3) η=WrecWtotal×100%,

where $P_{m}$ is the saturation polarization, and $P_{r}$ the remnant polarization, E the external electric field, $W_{rec}$ the recoverable energy storage density, $W_{total}$ the total energy storage density, and η the energy storage efficiency. A good ESP should consist of at least two aspects, one is the high energy storage density, and another is the superior storage efficiency associated with the energy loss. More precisely, according to Equations (1)–(3), to achieve appropriate properties in energy storage, a large $\Delta P=P_{m}-P_{r}$ is required. In addition, a high breakdown electric field $E_{b}$ is also a critical parameter. Moreover, a smaller $P_{r}$ is not only associated with a larger discharge energy density but also a higher energy storage efficiency [5].

Polymer dielectrics have been intensively explored due to their ultrahigh dielectric breakdown strength (DBS) and large energy storage density at room temperature. The lack of temperature stability, however, remains a stumbling block for practical applications. Ceramic dielectrics are good alternatives for polymers owing to their excellent temperature stability. However, their DBSs are not as high as those of polymers, which have become a research focus. Currently, various ceramic dielectrics with multitudinous components have been investigated for decades. One typical category is lead-based ceramics, which were regarded as the most potential materials for power devices because of their high polarization and double hysteresis loops, a nature of antiferroelectric. Although Liu et al. obtained an ultrahigh $W_{rec}$ of 11.2 J/cm^3^ with a decent η of 82% in (Pb_0.94_La_0.02_Sr_0.04_)(Z_r0.9_Sn_0.1_)_0.995_O_3_ thick film in 2019, the lead element raised numerous environmental issues, severely hindering the development of applications [6]. Thus, lead-free ceramics have been paid much attention, e.g., BaTiO_3_ (BT)-, Na_0.5_Bi_0.5_TiO_3_ (NBT)-, (K_0.5_Na_0.5_)NbO_3_ (KNN)-, BiFeO_3_ (BF)-, AgNbO_3_ (AN)-based ceramics, etc. Square-like P-E hysteresis loop and lower saturation polarization (~26 μC/cm^2^) in pure BT ceramics may cause poor ESP. Although ultrahigh saturation polarization (~100 μC/cm^2^) was found in tetragonal pure BF ceramics [7], a large leakage current resulting from the variable valance state of iron ions inevitably limits the appearance of such a large polarization, and then the applications of BF-based ceramics in energy storage. In addition, the high cost of Nb-based ceramics, e.g., KNN-based and AN-based ceramics, makes them not conducive to industrialization. In comparison to the materials systems mentioned above, a typical lead-free material, NBT-based ceramic, stands out and presents promising potential in energy storage applications due to its large saturation polarization (more than 40 μC/cm^2^), resulting from the orbital hybridization of Bi^3+^ 6p and O^2-^ 2p, slim P-E hysteresis loops due to the nano polar region structure, and operational stability [3]. Pure NBT ceramic, however, is unsuitable for energy storage applications owing to its large $P_{r}$, high coercive field, and low DBS, thus usually, structural, and compositional designs are used to optimize the hysteresis loops and DBS of NBT-based material systems [8]. The most common method in compositional design is the ionic doping or forming of solid solution, e.g., (BaSr)TiO_3_ in terms of SrTiO_3_ (ST) and BT [9,10], which can effectively improve the ESP via breaking the relevance of adjacent dipoles and forming the polar nanoregions (PNRs).

As a novel relaxor ferroelectric, Sr_0.7_Bi_0.2_TiO_3_ (SBT) exhibits a significantly diffuse phase transition over a wide temperature range and possesses high polarization due to the substitution of Bi^3+^ ions to part of Sr^2+^ ions in ST. A $W_{rec}$ of 3.1 J/cm^3^ with a η of 93% was achieved at 360 kV/cm in 0.9(Sr_0.7_Bi_0.2_)TiO_3_–0.1Bi(Mg_0.5_Hf_0.5_)O_3_ ceramics [11]. Whereafter, a high $W_{rec}$ of 3.03 J/cm^3^ with a η of 79.5% was reported by Wang et al. for 0.95[0.7(Na_0.5_Bi_0.5_)TiO_3_-0.3(Sr_0.7_Bi_0.2_)TiO_3_]-0.05Bi(Mg_0.5_Ti_0.5_)O_3_ ceramics [12]. It can be revealed that a certain amount of SBT can increase the DBS and enhance the relaxor ferroelectric characteristics, thus improving the ESP. Meanwhile, another kind of dopant, i.e., Bi(M_1_M_2_)O_3_ (M_1_ = Mg^2+^, Zn^2+^ etc., M_2_ = Ti^4+^, Nb^5+^, Zr^4+^, Hf^4+^ etc.) [13,14,15], has been widely employed in compositional optimization, e.g., BT-Bi(M_1_M_2_)O_3_, NBT- Bi(M_1_M_2_)O_3_, NN- Bi(M_1_M_2_)O_3,_ etc., due to its significant impact on the improvement of DBS and lowering of the $P_{r}$ [16]. By doping Bi(Mg_1/3_Ta_2/3_)O_3_, the average DBS in (1−x) (0.6Na_0.5_Bi_0.5_TiO_3_-0.4Sr_0.7_Bi_0.2_TiO_3_)-xBa(Mg_1/3_Ta_2/3_)O_3_ ceramics increases from 230 kV/cm to 570 kV/cm [17], indicating that Bi(M_1_M_2_)O_3_ dopants can significantly increase the ESP.

In this work, a compositional tailoring strategy was employed to attain superior ESP. Doping with SBT and Bi(Mg_0.5_Zr_0.5_)O_3_ (BMZ) can not only enhance the relaxation degree by breaking the long-range ferroelectric order and forming the PNRs, but also improve the DBS. To achieve this strategical objective, the (1−x)(0.7Na_0.5_Bi_0.5_TiO_3_-0.3Sr_0.7_Bi_0.2_TiO_3_)-xBi(Mg_0.5_Zr_0.5_)O_3_ (NBT-SBT-xBMZ) (x = 0, 0.09, 0.12, 0.15 and 0.18) ceramics were successfully fabricated using a solid-state reaction method. An enhanced ESPs, e.g., a high *W_rec_* of 6.75 J/cm^3^ and a *η* of 79.44% along with an ultrafast discharge time of ~49.1 ns, were obtained in the sample of x = 0.15. Furthermore, excellent stabilities of energy storage density versus temperature and frequency were procured for the ceramics with the same composition, providing a potential material candidate for practical applications in advanced power pulse devices.

## 2. Materials and Methods

The (1−x)(0.7Na_0.5_Bi_0.5_TiO_3_-0.3Sr_0.7_Bi_0.2_TiO_3_)-xBi(Mg_0.5_Zr_0.5_)O_3_ (abbreviated as NBT-SBT-xBMZ) (x = 0, 0.09, 0.12, 0.15 and 0.18) ceramics were fabricated using a conventional solid-state reaction approach. The raw materials of Na_2_CO_3_ (>99.8%, Aladdin, Shanghai, China), Bi_2_O_3_ (>99.0%, Aladdin, Shanghai, China), TiO_2_ (>99.0%, Aladdin, Shanghai, China), SrCO_3_ (>99.0%, Aladdin, Shanghai, China), ZrO_2_ (>99.0%, Aladdin, Shanghai, China), and MgO (>98.0%, Aladdin, Shanghai, China) were weighted in accordance with the stoichiometry. After mixing with ethanol solvent and ZrO_2_ balls, the powders were uniformly mixed in a plastic bottle and ball-milled at 250 rpm for 12 h in a planetary ball miller. Afterwards, the slurry was dried at 60 °C for 10 h. To obtain NBT-SBT-xBMZ perovskite crystallites, the powders were grinded, sieved, and calcined at 850 °C for 4 h. Then, 5 wt% polyvinyl butyral (PVB) was used as a binder and the granulated powders were pressed into disks with 10 mm in diameter and ~1 mm in thickness under an axial pressure of 6 MPa. Whereafter, isostatic pressing at 200 Mpa was pursued for further pre-densification. Ultimately, the disks were sintered in the temperature range from 1140 to 1180 °C for 4 h after burnout of PVB. To avoid the vaporization of Bi^3+^, the as-prepared pellets were buried sintered.

The polycrystalline structure of the NBT-SBT-xBMZ was measured using X-ray diffractometry (XRD, Rigaku Ultima IV diffractometer) with the Cu Kα radiation. The microtopography of various specimens was characterized using a scanning electron microscope (SEM, Hitachi S-3400N) and the grain size distributions were statistically analyzed employing ImageJ software. Temperature-dependent dielectric properties and impedance properties were procured using a dielectric property measurement system (DMS-500, Partulab, Wuhan, China and E4980A, Agilent, Santa Clara, CA, USA) at 1, 10, 100 kHz, and 1 MHz from 30 °C to 350 °C with a heating rate of 3 °C/min. A Sawyer–Tower circuit (The Precision Premier II, Radiant Technologies, Inc., Albuquerque, NM, USA) was employed to measure the P-E hysteresis loops and current-electric field (I-E) loops. A DC high voltage with a rising rate of 0.1 kV/s was supplied to the sample using a high voltage signal source (Model 610E, Trek, Inc., Lockport, New York, NY, USA). The charge and discharge performance of the ceramics was measured using a well-designed charge/discharge circuit. The ceramics were polished down to 0.1 mm in thickness and sputtered with gold electrodes of 2 mm in diameter on each side were prepared for all electrical measurements. During the electrical measurements, the samples were put into silicone oil to avoid electric breakdown.

## 3. Results and Discussion

### 3.1. Phase Structure and Microstructure

**Figure 1:**
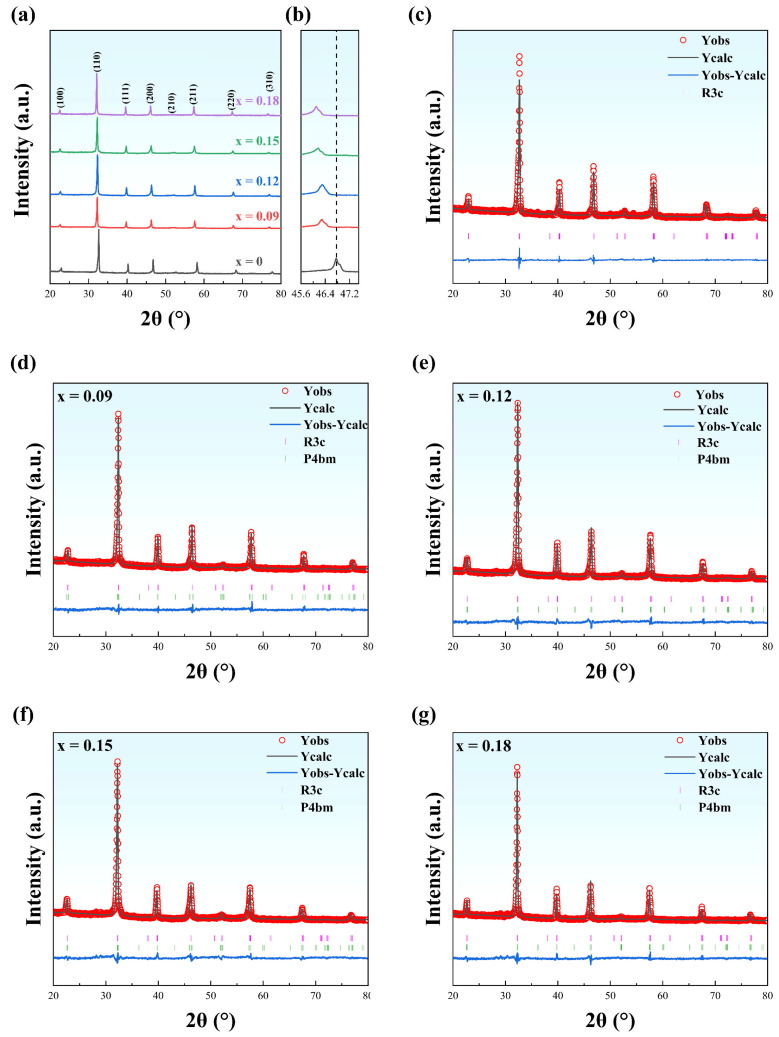
XRD patterns and Rietveld refinements for (1−x)(0.7Na_0.5_Bi_0.5_TiO_3_-0.3Sr_0.7_Bi_0.2_TiO_3_)-xBi(Mg_0.5_Zr_0.5_)O_3_ (NBT-SBT-xBMZ) ceramics. (**a**) XRD patterns; (**b**) magnified XRD patterns near 46°; (**c**–**g**) Rietveld refinements for samples of x = 0, 0.09, 0.12, 0.15, and 0.18, respectively.

The room-temperature XRD patterns for NBT-SBT-xBMZ specimens are presented in Figure 1a. One can observe that all compositions display a pure polycrystalline perovskite structure without a trace of a secondary phase, indicating that multi-cations have completely entered into the perovskite lattice sites, e.g., Mg^2+^, Zr^4+^, and Sr^2+^ sites. In addition, the peak near 46° shifts towards low diffraction angles, disclosing the lattice expansion due to the substitution of ions with a larger ionic radius (here, R(Mg^2+^) = 0.072 nm and R(Zr^4+^) = 0.072 nm) for a smaller one (R(Ti^4+^) = 0.0605 nm). An asymmetric single-peak around 45° gradually evolves to a weak split-peak, as shown in Figure 1b, implying the multi-phase coexistence, which can be further confirmed using the Rietveld refinement (see Figure 1c–g). Similar to other NBT-based relaxor ferroelectrics [16,18], rhombohedral phase (R phase, space group *R3c*), and tetragonal phase (T phase, space group *P4bm*) coexist for all specimens doped with BMZ at room temperature. As shown in Table 1, all the reliability factors of the weighted pattern (*R_wp_*) and chi-square (*χ^2^*) are less than 7.5% and 4, respectively, suggesting a good agreement between the measured results and the refined results using R phase and T phase two-phase mode. Furthermore, when the BMZ content increases, there is a rise in T phase fraction and a drop in R phase.

To further explore the relationship between the BMZ content and the phase fraction evolved, Raman spectroscopy (see Figure S1) was employed. Based on the published data [19,20], the Raman spectrum of NBT-SBT-xBMZ ceramics can be divided into four regions within 100~1000 cm^−1^ in terms of the corresponding vibration modes. The regions ranging from 50 to 200, from 200 to 400, from 400 to 700, and >700 cm^−1^ are related to the vibration mode of A-site cations, B-O bond, oxygen octahedron (BO_6_), and A1 + E bonds, respectively [20]. Compared with the sample of x = 0, other samples doped with BMZ present more diffuse Raman peaks in the range of 50~200 and 200~400 cm^−1^, implying an enhanced disorder in both A and B sites. Meanwhile, the multi-ionic doping also leads to the increase of oxygen octahedral tilting, which can be verified by the Raman peaks, ranged from 400 to 700 cm^−1^ [21]. All results indicate that the short-range disorder has been enhanced via multi-ionic doping, resulting in the induced PNRs and further improved ESP [16].

**Figure 2:**
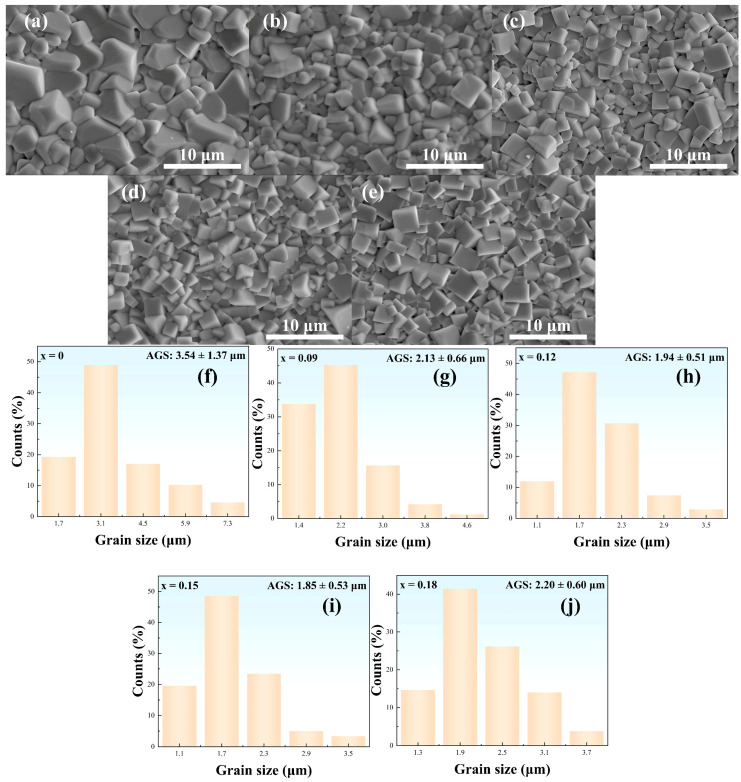
Surface morphologies of NBT-SBT-xBMZ ceramics. (**a**–**e**) Scanning electron microscope (SEM) images of various compositions; (**f**–**j**) the corresponding statistics of grain size distributions of the SEM images (**a**–**e**).

The surface morphologies for NBT-SBT-xBMZ specimens are shown in Figure 2a–e. All specimens exhibit distinct grain boundary and dense grains without visible pores. The corresponding average grain sizes (AGS) shown in Figure 2f–j are statistically calculated using the ImageJ program. One can observe that the AGS for NBT-SBT-xBMZ specimens first decreases from 3.54 ± 1.37 μm (x = 0) to 1.85 ± 0.53 μm (x = 0.15), and then increases to 2.20 ± 1.37 μm when x further increases (x = 0.18 in this case), which indicates that the grain boundary migration can be inhibited and the AGS can be effectively decreased at appropriate BMZ content. This behavior can be attributed to the variation of lattice strain energy (Δ*G_strain_*), which can be determined by Equation (4) [22].

(4) $$\Delta G_{strain}=4\pi MN_{A}[\left(r_{0}/2\right){\left(r_{d}-r_{0}\right)}^{2}+\left(1/3\right)\left({\left(r_{d}-r_{0}\right)}^{3}\right],$$

where *N_A_* is the Avogadro constant, *M* the Young’s modulus, *r_d_* the doped ionic radius and *r_0_* the optimal radius of the lattice site. Owing to the large doped ionic radius (R(Mg^2+^) = 0.072 nm and R(Zr^4+^) = 0.072 nm), the change in Δ*G_strain_* resulting from the radius mismatch becomes increasingly large and then inhibits the grain boundary migration for appropriate BMZ contents, leading to a decrease in grain size. The fine grain size and compact structure might be beneficial to the ESP.

### 3.2. Dielectric Properties

**Figure 3:**
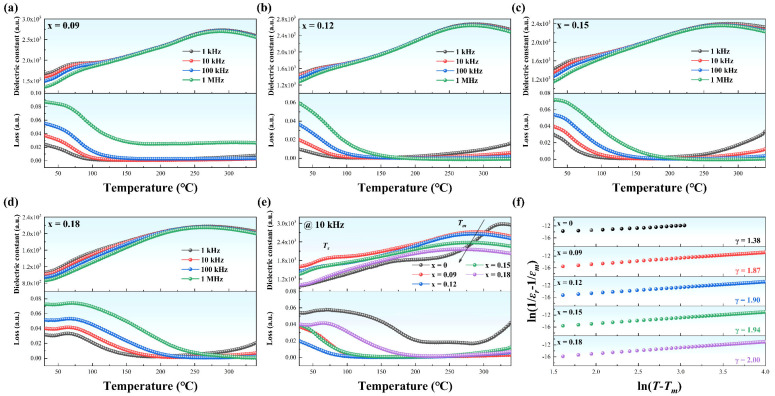
Temperature-dependent dielectric properties for NBT-SBT-xBMZ ceramics in the temperature range from 30 to 340 °C. (**a**) x = 0.09; (**b**) x = 0.12; (**c**) x = 0.15; (**d**) x = 0.18; (**e**) dielectric constant and loss tangent as a function of temperature for various compositions at 10 kHz; (**f**) ln(1/*ε_r_* − 1/*ε*) as a function of ln(*T* − *T_m_*).

The dielectric properties as a function of temperature and frequency for NBT-SBT-xBMZ ceramics measured in the temperature range from 30 to 340 °C at 1 kHz, 10 kHz, 100 kHz, and 1 MHz are shown in Figure 3a–d and Figure S2. Compared with the pure NBT-SBT with a weak relaxor ferroelectric characteristic, the specimens doped with BMZ reveal stronger relaxor ferroelectric characteristics, i.e., more distinct frequency dispersion and a broader phase transition temperature regime. In addition, two anomalies, including the low-temperature shoulder (*T_s_*) and high-temperature peak (*T_m_*), are found in all specimens. Accordingly, the low-temperature one is caused by the thermal evolution of *R3c* and *P4bm* PNRs, while the high-temperature one is related to the phase transition from *R3c* to *P4bm* PNRs and the thermal evolution of *P4bm* PNRs [23]. As revealed in Figure 3e, the *T_m_*s shift towards low temperatures from 329 to 266 °C with the increasing BMZ content, implying higher T phase content may be obtained at a certain temperature, which is consistent with the results of XRD refinement shown in Table 1. Especially for the specimens doped with BMZ, the *T_m_*s decrease at a sluggish rate. Meanwhile, the $\varepsilon_{m}s$ at *T_m_*s gradually decrease, and the permittivity curves become smooth, which is conducive to obtaining a high DBS and excellent temperature stability [24].

Generally, the diffuseness for some relaxor ferroelectrics can be estimated using the modified Curie–Weiss law expressed in Equation (5),

(5) $$\frac{1}{\varepsilon }-\frac{1}{\varepsilon_{m}}=\frac{{\left(T-T_{m}\right)}^{\gamma }}{C}\;,$$

where *T* is temperature above *T_m_*, ε the dielectric constant at *T*, *C* the Curie constant. The exponent γ, the value of which is between 1 (normal ferroelectrics) and 2 (relaxor ferroelectrics), is used to describe the diffuseness degree. As presented in Figure 3f, the diffuseness degree significantly increases from 1.38 (x = 0) to 2.00 (x = 0.18) by doping BMZ, resulting from the breaking of the correlation of adjacent dipoles and constructing of PNRs [25]. Note that the γ for the specimens doped with BMZ are 1.87, 1.90, 1.94, and 2.00, respectively, indicating that the behaviors close to relaxor ferroelectrics are expected in all samples, which plays a critical role in decreasing *P_r_*, improving *η*, and enhancing the thermal stabilities, thus an enhanced ESP can be attained.

### 3.3. Ferroelectric and ESP

**Figure 4:**
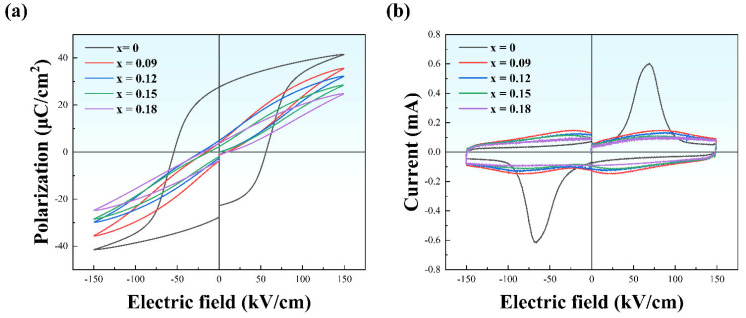
(**a**) Bipolar P-E loops and (**b**) bipolar I-E loops under 150 kV/cm at 100 Hz for various compositions of NBT-SBT-xBMZ ceramics (x = 0, 0.09, 0.12, 0.15, 0.18).

Figure 4a presents the bipolar P-E hysteresis loops of NBT-SBT-xBMZ ceramics (x = 0, 0.09, 0.12, 0.15, and 0.18) at 150 kV/cm and 100 Hz. The sample of x = 0 exhibits strong ferroelectric characteristics with a large *P_m_* (41.48 μC/cm^2^) and *P_r_* (27.65 μC/cm^2^), which is unfavorable for ESP. Note that the P-E loops for the specimens doped with BMZ become slimmer. Specifically, the *P_m_* slightly decreases while the *P_r_* significantly decreases at the same electric field of 150 kV/cm. We propose a possible reason for the decrease in *P_m_* at the same electric field with the increase of BMZ. The rise in weakly polar T phase fraction is accompanied by a drop in a strongly polar R phase, which reduces the *P_m_* to a certain extent. This can be verified by the Rietveld refined results listed in Table 1. The current density-electric field (I-E) loops at 150 kV/cm and 100 Hz are depicted in Figure 4b. Two prominent current peaks correspond to the switching of ferroelectric domains [26]. It should be noted that four current peaks were obtained in the samples doped with BMZ instead of two peaks, indicating a reversible field-induced phase transition, i.e., four compositions are in the ergodic phase. Furthermore, current peaks are gradually smooth with the increase of BMZ due to the closing to ideal relaxor ferroelectric characteristics (γ = 2), which is consistent with the results of Raman spectra and dielectric properties as a function of temperature and frequency.

**Figure 5:**
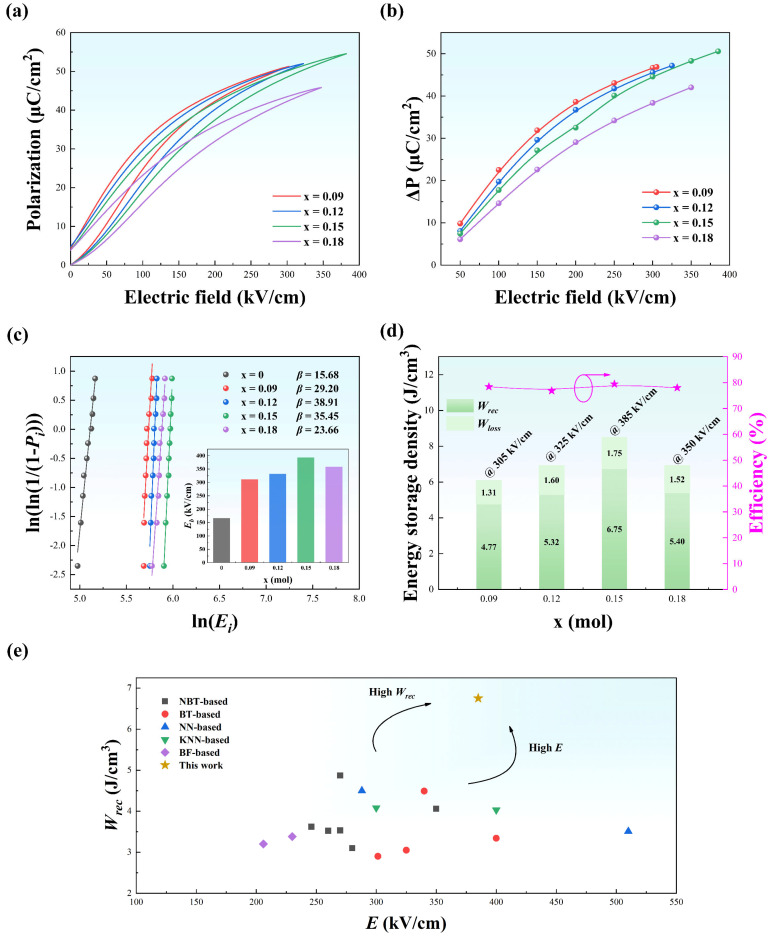
(**a**) Unipolar P-E loops at 100 Hz under various electric field; (**b**) the corresponding Δ*P* of (**a**); (**c**) the Weibull distribution; (**d**) the corresponding ESP of (**a**) for various composition of NBT-SBT-xBMZ ceramics (x = 0.09, 0.12, 0.15, 0.18); (**e**) comparison of recoverable energy storage densities and applied electric fields for the sample of x = 0.15 and other lead-free ceramics reported. Pink star’s meaning has been defined by the right *y*-axis, where the arrow is pointing.

The unipolar P-E loops measured at 100 Hz under various electric fields are shown in Figure 5a. In accordance with the dielectric properties, all specimens show the nature of typical relaxor ferroelectrics, i.e., all P-E loops are slim with small *P_r_*s. As mentioned above, the Δ*P* and DBS play a dominant role in affecting the ESP. The corresponding Δ*P*s procured by P-E loops and statistic DBS fitted using a Weibull distribution are illustrated in Figure 5b,c. Similar to the previous report [27], the delayed polarization saturation is attained via doping BMZ, leading to a larger *W_rec_*. Moreover, with the increasing applied electric field, the Δ*P*s continuously increases, resulting in the improved ESP, i.e., the *W_rec_* gradually increases and the *η* maintains ~80% for all compositions (see Figures S3 and S4). In addition, as revealed in Figure 5c, the DBS first increases (from 165.92 kV/cm @ x = 0 to 393.39 kV/cm @ x = 0.15), then decreases with the further increase of x (358.05 kV/cm @ x = 0.18), of which the tendency is opposite with the grain size. Hence, the highest value of DBS in x = 0.15 might be caused by the fewer vacancies, associated with the fine grain sizes and the dense structure. Furthermore, the slopes (*β*) of Weibull distribution are over 15, indicating the excellent stability of sample quality and high reliability of statistic DBS. Based on Equations (1)–(3), the relevant ESPs are indirectly procured, and shown in Figure 5d. Owing to the largest Δ*P* (50.58 μC/cm^2^) and the highest DBS (393.39 kV/cm) simultaneously achieved in the sample of x = 0.15, the optimal *W_rec_* of 6.75 J/cm^3^ with a *η* of 79.44% were also obtained for the same composition. Meanwhile, as revealed in Figure 5, the sample of x = 0.15 is superior to other reported lead-free ceramics [10,28,29,30,31,32,33,34,35,36,37,38,39,40,41,42] in both *W_rec_* and *E*, meaning a promising material system was successfully fabricated in this work.

**Figure 6:**
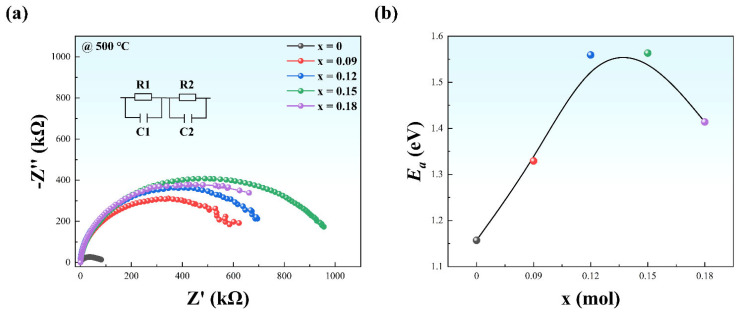
(**a**) The Nyquist plots (Z″ vs. Z′) measured at 100~2 MHz and 500 °C for NBT-SBT-xBMZ ceramics (x = 0, 0.09, 0.12, 0.15, 0.18); (**b**) the *E_a_* as a function of x.

Generally, the DBS of a material is determined by the intrinsic and extrinsic factors, in which the extrinsic factors are of dominance due to the ultrahigh intrinsic DBS (usually more than 1000 kV/cm), which outclasses the extrinsic ones. To explore the main reason for the optimal DBS occurred in the sample of x = 0.15 instead of 0.18, a complex impedance analyses were carried out. Figure 6a displays the Nyquist plots (Z″ vs. Z′) measured at 100~2 MHz and 500 °C for NBT-SBT-xBMZ ceramics (x = 0, 0.09, 0.12, 0.15, 0.18). It can be observed that a separate semicircle form presents in all impedance spectra, which indicates the same conductivity behaviors [43]. Specifically, for electroceramics, an equivalent circuit based on two parallel R and C elements in series contains grain resistance R1, grain capacitance C1, grain boundary resistance R2, and grain boundary capacitance C2, can be utilized to model the impedance results, as shown in the inset of Figure 6a [16]. The fitted results for the resistances and capacitances of grain and grain boundary are shown in Table 2. It is observed that the resistance of grain boundary outclasses that of the grain, indicating the dominant contribution of the grain boundary. Meanwhile, the maximal R2 occurs in the sample of x = 0.15. Obviously, the semicircles become tremendously enlarged by doping BMZ, implying a significant enhancement in resistance. Furthermore, as revealed in Figure 6a, the semicircles first become large and then small with the increase of BMZ, which is consistent with the trend of resistances and Weibull distributions as a function of x. The activation energy (*E_a_*) for the conductivity can be calculated based on the Arrhenius relation (Equation (6)) [44]

(6) $$\sigma =\sigma_{0}\exp\left(E_{a}/k_{B}T\right)\;,$$

where σ is the conductivity, $\sigma_{0}$ the pre-constant, $k_{B}$ the Boltzmann constant, T the absolute temperature. The *E_a_* of NBT-SBT-xBMZ ceramics is depicted in Figure 6b. One can see that the values of *E_a_* for all specimens are close to 1, indicating the conductivity at high temperatures is related to the oxygen vacancies [45]. Note that *E_a_* increases from 1.16 (@ x = 0) to 1.56 (@ x = 0.15) and then decreases to 1.41 (@ x = 0.18), revealing a similar tendency for oxygen vacancies with different BMZ contents. For the vacancies that exist in ceramics, firstly, both the oxygen vacancies and the bismuth vacancies exist. Secondly, the oxygen vacancy concentration is usually determined by the oxygen partial pressure, dopants, cooling rate from the sintering temperature, etc. Since these conditions are almost the same for four samples, the oxygen vacancy concentration can be regarded as almost the same with different BMZ contents. Thirdly, the incorporation of BMZ may produce Bi^3+^ vacancies as a result of the evaporation of Bi^3+^ ions during the sintering at high temperatures, which will compensate for the oxygen vacancies since their electrical signs are opposite. The bismuth vacancy concentration increases with the BMZ content. Fourthly, except for x = 0.18, which might contain too large bismuth vacancy concentration to be completely compensated by the oxygen vacancies, the oxygen vacancies dominate before x = 0.15, and the resistance increases with the increasing BMZ content, i.e., x value. At x = 0.15, the oxygen vacancy concentration is almost the same as that of bismuth vacancy, resulting in the largest impedance shown in Figure 6. Therefore, the increased impedance and the decreased vacancy concentrations are conducive to the large DBS of the ceramics.

Beyond that, the AGS also makes a critical impact on the DBS and is inversely proportional to the DBS according to Equation (7) [1].

(7) $$DBS\;\propto G^{-a}\;,$$

where a is an exponent ranging from 0.2 to 0.4. As mentioned above, the sample of x = 0.15 has the smallest AGS, demonstrating that a higher DBS might be achieved with the same composition.

It can be concluded that the fine grain sizes, dense microstructure, smooth current peak, higher resistance, increased *E_a_*, and diminished oxygen vacancy concentration collectively contribute to the improved DBS.

**Figure 7:**
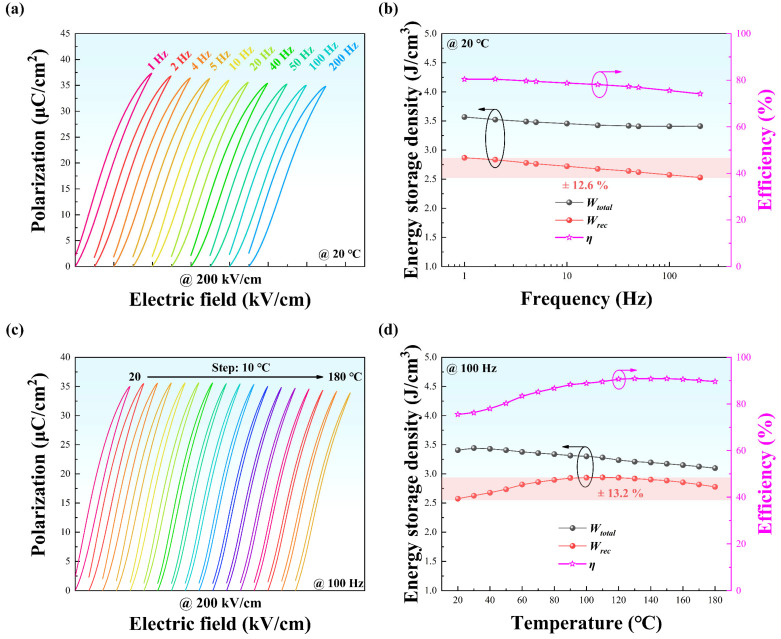
(**a**) Frequency-dependent unipolar P-E loops of x = 0.15 sample at 20 °C under 200 kV/cm in the frequency range of 1–200 Hz; (**b**) the corresponding ESP of (**a**); (**c**) temperature-dependent unipolar P-E loops of x = 0.15 sample at 100 Hz under 200 kV/cm in the temperature range of 20–180 °C; (**d**) the corresponding ESP of (**c**).

It is essential to investigate the stabilities of frequency and temperature for sample of x = 0.15, which can help confirm the applicable working temperature range and frequency range. The frequency-dependent and temperature-dependent unipolar P-E loops are shown in Figure 7a,c. Meanwhile, the corresponding ESP calculated based on Equations (1)–(3) are depicted in Figure 7b,d, respectively. All specimens show slim P-E loops at various frequencies and temperatures. Moreover, with the reduction of frequency, the P-E loops become slimmer, resulting in an increase in *η* and *W_rec_*. As a result, only a variation of 12.6% in *W_rec_* can be observed in the frequency range of 1–200 Hz, indicating good frequency stability. Interestingly, the P-E loops become significantly slim when the temperature exceeds ~60 °C, which is closed to the *T_s_* for the sample of x = 0.15. It can be attributed to the ergodic relaxor ferroelectric state, in which the PNRs formed by doping BMZ can convert into long-range ferroelectric order under the application of a strong enough electric field, and convert back to the ergodic state, resulting in the maintained large *P_m_* and dramatically decreased *P_r_* [46]. Especially, despite a large variation in *η* from 75.6% (20 °C) to 90.92% (110 °C), only ±13.2% variation is detected in a temperature range from 20 to 180 °C, implying a good thermal stability. One can conclude that the sample of x = 0.15 possesses good thermal and frequency stabilities simultaneously. Beyond that, the *T_s_* can be shifted to lower temperatures via compositional tailoring, providing a method to obtain the superior ESP near room temperature.

Moreover, the charge-discharge properties are also of importance to practical applications. Theoretically, the current density (*C_D_*), power density (*P_D_*), discharge speed, and discharged energy storage density (*W_d_*) can be procured according to Equations (8)–(10) [47]. Especially the discharge time (*t*_0.9_) is evaluated by the time when *W_d_* is up to 90% of the maximal value under an electric field.

(8) $$C_{D}=I_{max}/S\;,$$

(9) $$P_{D}=EI_{max}/2S\;,$$

(10) $$W_{d}=R\int i{\left(t\right)}^{2}dt/V\;,$$

where *I_max_* is the maximal current peak in undamped *i*(*t*) ~ *t* curves, *S* the effective electrode area, *V* the effective volume of sample, *E* the applied electric field, *R* the load resistance (100 Ω in this work) and *i(t)* the discharge current in overdamped curves.

**Figure 8:**
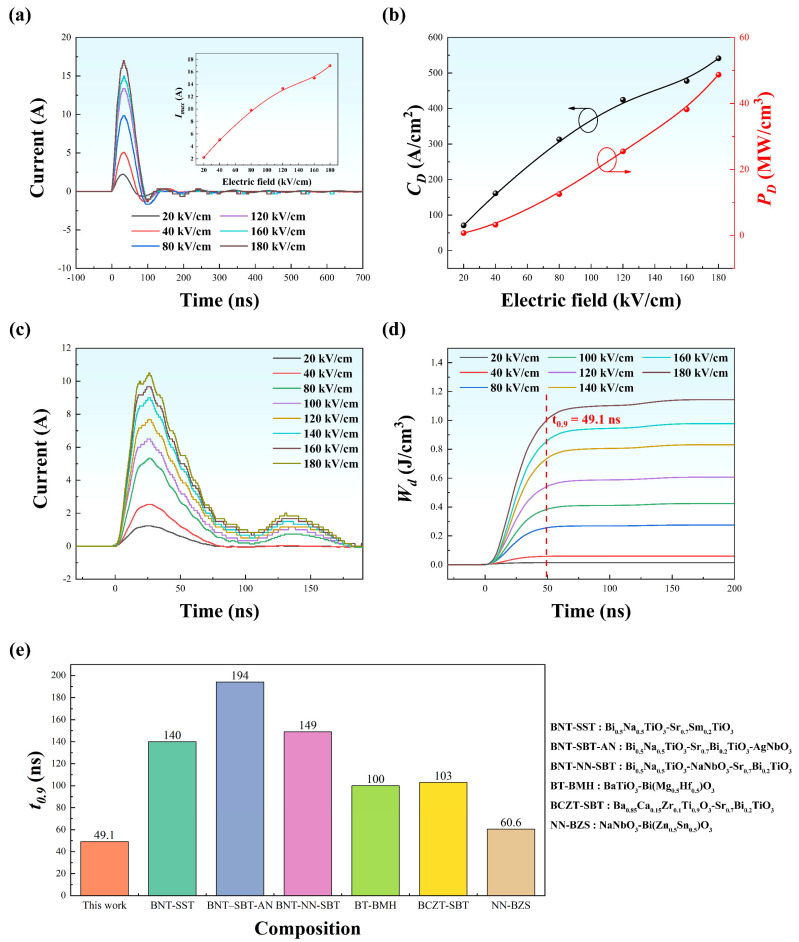
(**a**) Undamped discharge waveform, and (**b**) variation of *C_D_*, *P_D_* at various electric fields for x = 0.15 sample; (**c**) overdamped discharge waveform; (**d**) time-dependent discharge energy density at various electric fields for x = 0.15 sample; (**e**) comparison of *t*_0.9_ for the sample of x = 0.15 and other lead-free ceramics recently reported.

Therefore, the charge-discharge properties for the sample of x = 0.15 were evaluated for its performance in a circuit. The undamped discharge current curves under various electric fields (20–180 kV/cm) are displayed in Figure 8a. The inset shows the variation of *I_max_*, suggesting that the highest *I_max_* reaches 17 A under 180 kV/cm. Meanwhile, as revealed in Figure 8b, a high *C_D_* of 541.40 A/cm^2^ and *P_D_* of 48.73 MW/cm^3^ are simultaneously achieved at the same electric field. Figure 8c presents the overdamped discharge waveforms under various electric fields (20–180 kV/cm).

Based on the classic RLC circuit, the discharge process can be modeled using a second-order differential equation of current as a function of time. Basically, the solution of the equation is composed of an exponential function and a sinusoidal function. The former determines the amplitude of the discharged current, while the latter determines the periodical oscillation of the discharged current, depending on the load, i.e., the resistance. If the resistance is smaller, e.g., R << 2$\sqrt{L/C}$, the decay of the discharged current is not prominent, the oscillation is dominant. This process is usually called under-damped process. However, if the resistance is larger, the decay of discharged current becomes significant, and the oscillation of discharged current becomes unimportant, but it does exist to some extent. This is what we observed in Figure 8c [48].

One can see that *t*_0.9_ slightly increases along with the increase of electric field and reaches ~49.1 ns under 180 kV/cm, implying an ultrafast discharge time. In addition, a *W_d_* of 1.16 J/cm^3^ was calculated based on the overdamped discharge waveform according to Equation (10). As presented in Figure 8e, the discharge time *t*_0.9_ of ~49.1 ns in our work is superior among the published materials [13,28,29,49,50,51], meaning its great potential in advanced power pulse devices.

## 4. Conclusions

In conclusion, novel lead-free (1−x)(0.7Na_0.5_Bi_0.5_TiO_3_-0.3Sr_0.7_Bi_0.2_TiO_3_)-xBi(Mg_0.5_Zr_0.5_)O_3_ (NBT-SBT-xBMZ) relaxor ceramics were successfully fabricated using a solid-state reaction method and designed via compositional tailoring. Appropriate Bi(Mg_0.5_Zr_0.5_)O_3_ content can effectively enhance the relaxor ferroelectric behaviors and improve the DBS by forming fined grain sizes, increasing resistance, and diminishing oxygen vacancy concentrations. Therefore, the optimal *W_rec_* of 6.75 J/cm^3^ and a *η* of 79.44% were simultaneously obtained in NBT-SBT-0.15BMZ at 20 °C and 385 kV/cm. Meanwhile, a good thermal stability (20–180 °C) and frequency stability (1–200 Hz) associated with the ultrafast discharge time of ~49.1 ns were also obtained in the same composition, providing a promising material system for applications in power pulse devices.

## Figures and Tables

**Table 1:** The lattice parameters and R-factors obtained using the Rietveld refinement.

x	Space Group	Phase Fractions	Lattice Parameters (Å)			Volume (Å^3^)	R-Factors		
			*a*	*b*	*c*		*R_p_*(%)	*R_wp_*(%)	*χ* ^2^
0	*R3c*	100.00	5.48944	5.48944	13.41966	350.209	6.27	8.10	4.29
0.09	*R3c*	92.63	5.53055	5.53055	13.53951	358.650	4.50	5.79	2.65
	*P4bm*	7.37	5.51596	5.51596	3.94872	120.143			
0.12	*R3c*	82.67	5.53860	5.53860	13.55491	360.104	5.24	6.57	3.97
	*P4bm*	17.33	5.52976	5.52976	3.91932	119.846			
0.15	*R3c*	75.48	5.54761	5.54761	13.60827	362.698	5.88	7.32	3.73
	*P4bm*	24.52	5.53929	5.53929	3.94928	121.178			
0.18	*R3c*	60.33	5.55175	5.55175	13.58650	362.659	5.76	7.29	3.22
	*P4bm*	39.67	5.54833	5.54833	3.93289	121.070			

**Table 2:** Fitted values of equivalent circuit model for Nyquist plots.

Composition	R1 (Ω)	C1 (F)	R2 (Ω)	C2 (F)
x = 0	12,944	6.76 × 10^−10^	54,458	1.32 × 10^−9^
x = 0.09	177,600	8.90 × 10^−10^	501,600	9.05 × 10^−10^
x = 0.12	245,490	6.92 × 10^−10^	535,090	8.03 × 10^−10^
x = 0.15	98,490	3.29 × 10^−11^	850,890	5.19 × 10^−11^
x = 0.18	19,437	3.17 × 10^−10^	798,390	8.30 × 10^−10^

## Data Availability

Not applicable.

## Supplementary Materials

The following supporting information can be downloaded at: https://www.mdpi.com/article/10.3390/ma15175881/s1, Figure S1: Raman spectra for NBT-SBT-xBMZ ceramics; Figure S2: Dielectric constant and loss as a function of temperature and frequency for NBT-SBT-0BMZ ceramics in the temperature range of 30 to 340 °C; Figure S3: Unipolar P-E loops as a function of electric field at 100 Hz for (a) x = 0.09; (b) x = 0.12; (c) x = 0.15; (d) x = 0.18; Figure S4: The corresponding ESP as a function of electric field for P-E loops shown in Figure S3.
