# Supplementary material for: Enhanced Energy Storage Performance in Na0.5Bi0.5TiO3-Based Relaxor Ferroelectric Ceramics via Compositional Tailoring
Source: Materials (Basel). 2022 Aug 25;15(17):5881. doi: 10.3390/ma15175881 (PMC9457184; doi:10.3390/ma15175881)
Supplement: Supplementary file 1 [file materials-15-05881-s001.zip › materials-1843726-supplementary.pdf]

Supporting information

Enhanced Energy Storage Performance in $Na_{0.5}Bi_{0.5}TiO_3$-Based Relaxor Ferroelectric Ceramics via Compositional Tailoring

Yuleng Jiang [1,†], Xiang Niu [1,†], Wei Liang [1], Xiaodong Jian [1,*], Hongwei Shi [2], Feng Li [2], Yang Zhang [1], Ting Wang [3], Weiping Gong [3], Xiaobo Zhao [1], Yingbang Yao [1], Tao Tao [1], Bo Liang [1] and Shengguo Lu [1,4,5,*]

1 Guangdong Provincial Research Center on Smart Materials and Energy Conversion Devices, Guangdong Provincial Key Laboratory of Functional Soft Condensed Matter, School of Integrated Circuits, School of Materials and Energy, Guangdong University of Technology, Guangzhou 510006, China
2 Information Materials and Intelligent Sensing Laboratory of Anhui Province, Institutes of Physical Science and Information Technology, Anhui University, Hefei 230601, China
3 Guangdong Provincial Key Laboratory of Electronic Functional Materials and Devices, Huizhou University, Huizhou 516001, China
4 School of Integrated Circuits, Guangdong University of Technology, Guangzhou 510006, China
5 Dongguan South China Design Innovation Institute, Dongguan 523808, China
* Correspondence: jianxiaodong@gdut.edu.cn (X.J.); sglu@gdut.edu.cn (S.-G.L.)
† These authors contributed equally to this work

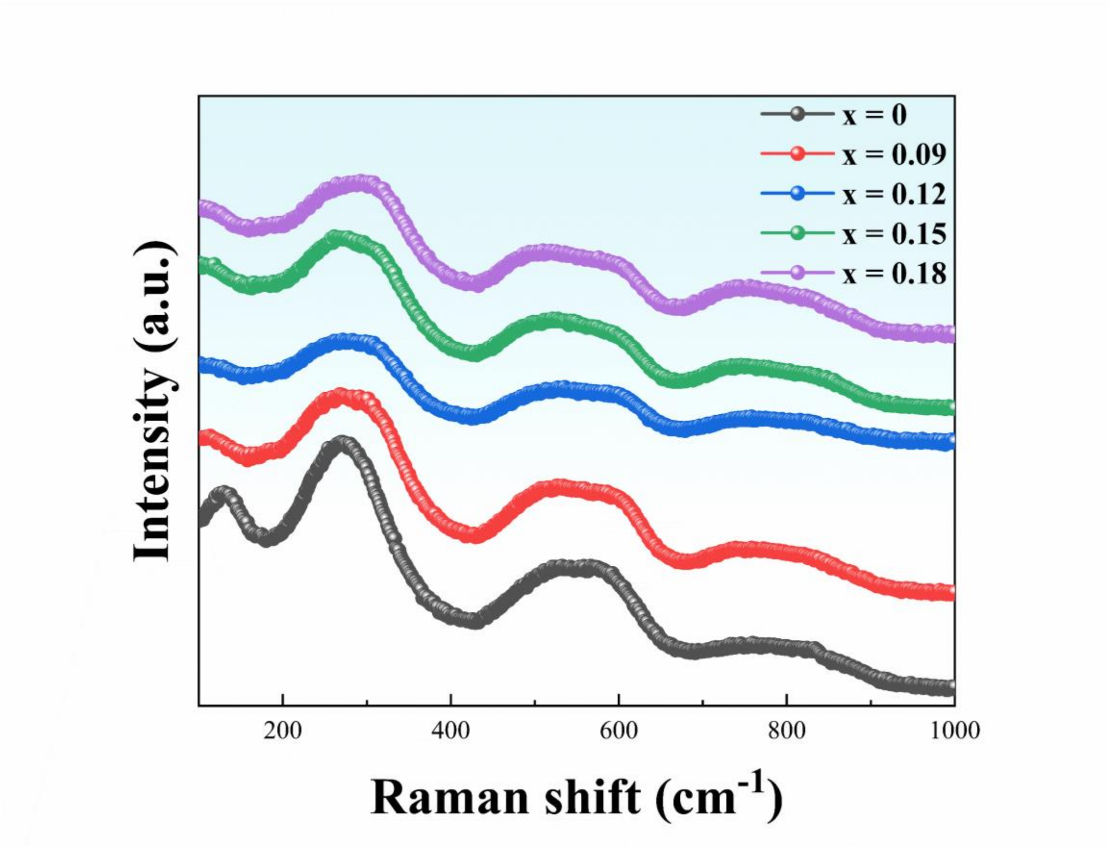

Figure S1. Raman spectra for NBT-SBT-xBMZ ceramics.

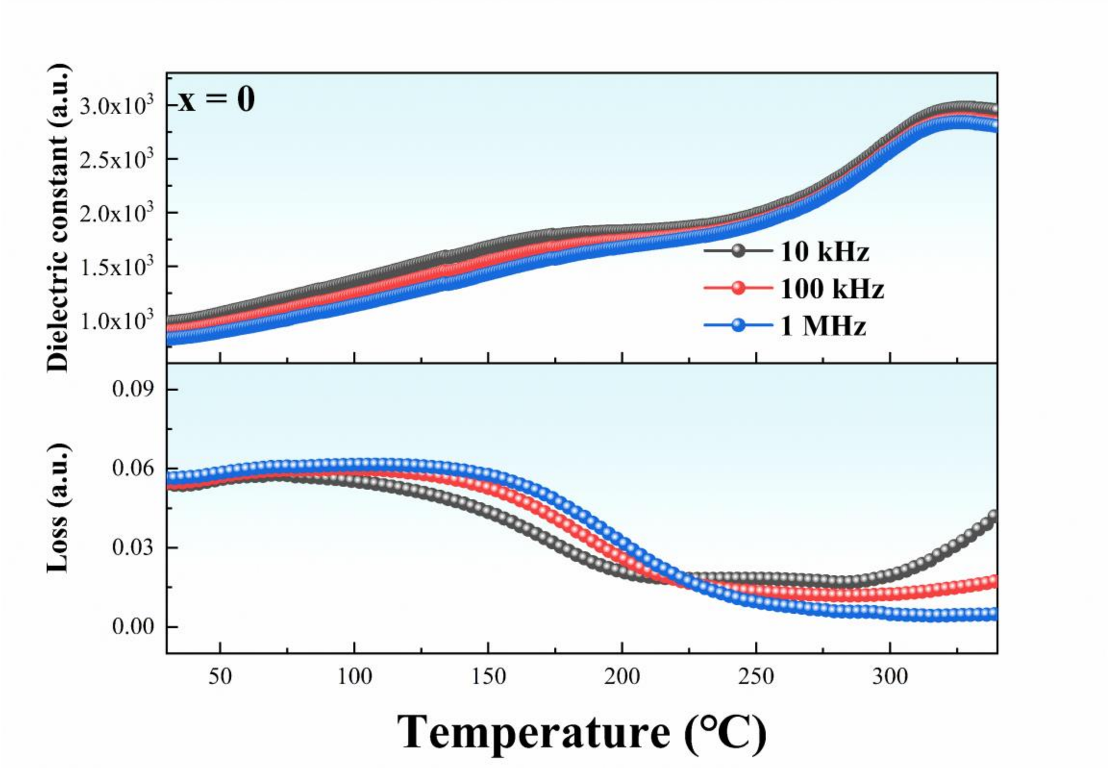

Figure S2. Dielectric constant and loss as a function of temperature and frequency for NBT-SBT-0BMZ ceramics in the temperature range of 30 to 340 °C.

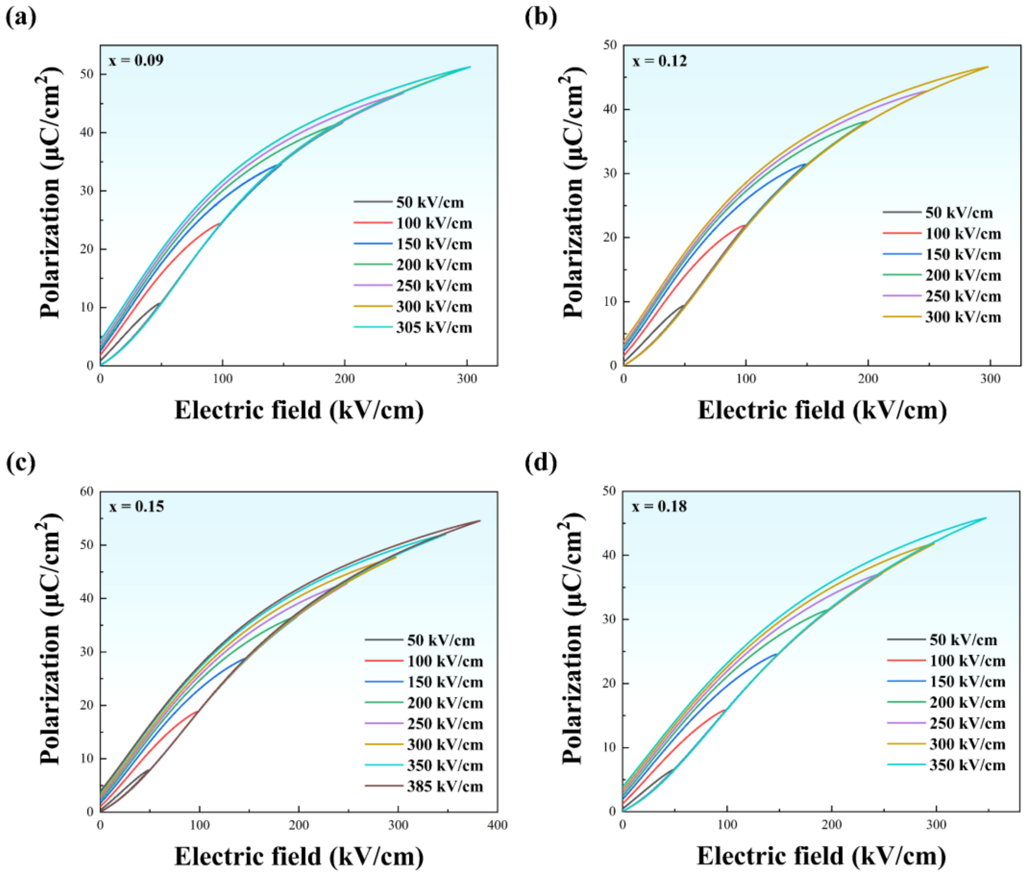

Figure S3. Unipolar P-E loops as a function of electric field at 100 Hz for (a) x = 0.09; (b) x = 0.12; (c) x = 0.15; (d) x = 0.18.

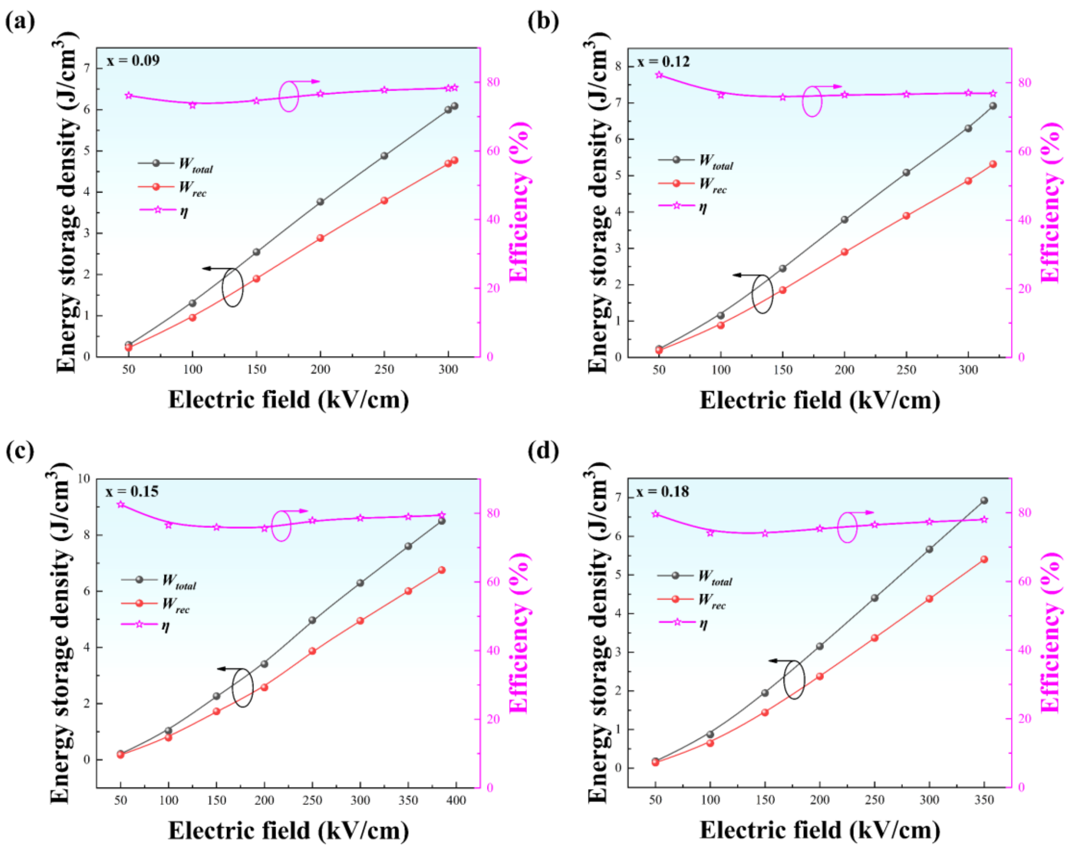

Figure S4. The corresponding ESP as a function of electric field for P-E loops shown in Figure S3.
